# Reimagining lung screening in women

**DOI:** 10.1093/bjr/tqaf134

**Published:** 2025-06-16

**Authors:** Kim Lori Sandler

**Affiliations:** Vanderbilt University Medical Center, Nashville, TN 37232-2675, United States

Lung cancer is the leading cause of cancer-related mortality in women, killing more women annually in the United States than breast and ovarian cancers combined.^1^ Screening for lung cancer with low-dose CT (LDCT) saves lives by detecting lung cancer at its earliest and most treatable stages, reducing mortality from lung cancer by at least 20%.^2^^,^^3^ Lung screening may be even more effective in women, with one trial demonstrating a 69% reduction in lung cancer mortality in women with annual LDCT as compared to 6% in men.^4^ Screening, however, is currently only recommended for individuals with a smoking history,^5^ as screening recommendations are based primarily on randomized controlled trials that enrolled individuals with a significant history of smoking.^6^^,^^7^ Disturbing trends are emerging in lung cancer however, with an increased incidence of lung cancer in young women but not in young men that cannot be explained by tobacco use alone,^8^ suggesting that the determination of screening eligibility should be re-evaluated.

Lung cancer is a women’s health crisis and screening is not even available to many women diagnosed with this disease. As with any screening test, risks and benefits should be carefully considered as there are potential harms such as false positive results and overdiagnosis.^9^ Lung screening should be specifically evaluated for women at increased risk for lung cancer that is not related to tobacco exposure. While smoking remains the leading risk factor for lung cancer, there are several additional contributors, including environmental exposures, family history, genetic predisposition, and many others.^10^ Recent considerations include:

1. Specific gender differences in lung cancer development have been reported seen as a higher prevalence of lung cancer in young women as compared to young men.^11^^,^^12^
2. Women with a prior history of cancer are at increased risk for developing a second primary malignancy, which is most often lung.^13^
3. Women with a family history of lung cancer are at increased risk, particularly women of Asian descent.^14^

It has been well demonstrated in recent years that the landscape of lung cancer is evolving, with increasing cases in women as compared to men,^15^ with growing evidence that lung cancer may be a different disease in women as compared to men, especially for those who have never smoked.

Though lung cancer rates overall have been declining over the past 20 years, the female-to-male rate ratios have largely increased. This change cannot be fully explained by changes in smoking behaviour alone.^8^ We have long recognized the role of BRCA mutations in breast and ovarian cancer, and while there is not yet a named genetic predilection to lung cancer, BRCA1, BRCA2, and the tumour suppressor gene p53 have been proposed as being associated with adenocarcinoma of the lung.^16^ Exogenous hormones may also be contributory as a study of hormone replacement therapy found that women taking estrogen plus progestin for 10 years or longer had roughly a 50% greater incidence of lung cancer. This was after adjusting for confounding variables including age and smoking history.^17^

Additional data points to possible predisposition for lung cancer development not only with hormones but also with breast cancer. While lung cancer is the leading cause of mortality in women, breast cancer is the most frequently diagnosed cancer in women.^1^ Recent evaluation of over 2 million electronic health records suggests that these cancers may be linked, with a 2-fold increase in lung cancer diagnosis in women who are breast cancer survivors as compared to women without breast cancer.^18^ Data further suggest that women with hormone-receptor negative (aka triple negative) breast cancer are at even great risk,^19^ and that women who receive chemotherapy to treat breast cancer are at greatest risk as compared to those treated with radiation and/or endocrine therapy.

Genetic predisposition has also been demonstrated with an increased prevalence of lung cancer in women with a family history of the disease and no first-hand tobacco exposure. Meta-analysis has also shown the rate of LDCT-detected lung cancer in Asian women without a significant smoking history to be similar to that in men with a smoking history, and that LDCT reduces mortality in the group without first-hand tobacco exposure.^20^ While the majority of lung screening for women who have never smoked has been performed in Asia, a study in the United States has shown similar results. Preliminary reporting from the Female Asian Nonsmoker Screening Study (FANSS) demonstrated a lung cancer detection rate with LDCT in women of Asian descent who have never smoked to be greater than the National Lung Screening Trial (NLST). This suggests that personalized screening recommendations may be particularly beneficial in this population.^21^

Expansion of lung screening eligibility to women without a significant smoking history will require a re-examination of the risks and benefits of LDCT. One of the most commonly discussed obstacles to lung screening is the false discovery rate. The sensitivity of lung screening is excellent; however, the specificity is lower. Patients often require short-term follow-up and less often invasive testing for benign disease.^22^ Artificial intelligence and machine learning can improve both the characterization of screening-detected abnormalities and ultimately screening outcomes.^23^ Machine learning algorithm evaluation of imaging can also be combined with blood-based biomarkers to improve the early detection of disease. By combining radiomic evaluation of LDCT imaging and biomarkers that assist in cancer detection, the false positive rate of lung screening can be further reduced.

The future of lung screening will almost certainly include a multifactorial, personalized, approach, combining risk prediction models, machine learning, and blood-based biomarkers. Specific prediction models are already being developed to better assess lung cancer risk in women without a smoking history.^24^ It is imperative that we continue to focus our efforts on better understanding the incidence of lung cancer in women in order to improve early detection, potentially through the personalization of screening. This is not an easy task, not only due to the complexities of endogenous and exogenous contributors to lung cancer development but also due to the lack of awareness of lung cancer as a disease of women. A nationwide survey performed in 2024 revealed that only 10% of adults believed lung cancer was among the most likely cancers to affect women, while 35% believed it was among the most likely to affect men.^25^ An additional nationwide survey performed by the American Lung Association in 2020 revealed that only 8% of Americans recognized lung cancer as the leading cause of cancer-related mortality for women. Lung cancer research remains underfunded, receiving significantly less investment than many other cancer types including breast.^26^ This is despite the fact that mammography is much better utilized than LDCT for lung screening and that women screened for breast cancer are in fact dying from lung cancer.^27^ Recent efforts to combine breast and lung screening have been well-received by patients and should be expanded to improve enrolment of eligible women in lung screening programs.^28^

Women in medicine and particularly in imaging can lead the way in improving the early detection of lung cancer and saving more lives. Our efforts can be successfully tied to other women’s health services including mammography. It is more important now than ever before to bring attention and funding to the study of lung cancer in women as we see a shift in incidence that cannot be explained by smoking. Early detection of lung cancer is imperative for everyone, men and women, those with and without smoking histories, and our efforts in this population can ultimately improve outcomes for all lung cancer patients.
